# Effects of exclusive breastfeeding on educational attainment and longitudinal trajectories of grade progression among children in a 13-year follow-up study in Malawi

**DOI:** 10.1038/s41598-023-38455-5

**Published:** 2023-07-14

**Authors:** Shamsudeen Mohammed, Emily L. Webb, Clara Calvert, Judith R. Glynn, Bindu S. Sunny, Amelia C. Crampin, Estelle McLean, Shekinah Munthali-Mkandawire, Albert Lazarous Nkhata Dube, Fredrick Kalobekamo, Milly Marston, Laura L. Oakley

**Affiliations:** 1 Department of Non-communicable Disease Epidemiology, Faculty of Epidemiology and Population Health, London School of Hygiene & Tropical Medicine, Keppel Street, London, WC1E 7HT UK; 2 Department of Infectious Disease Epidemiology, Faculty of Epidemiology and Population Health, London School of Hygiene & Tropical Medicine, London, UK; 3 Centre for Global Health, Usher Institute, University of Edinburgh, Edinburgh, UK; 4 Department of Population Health, Faculty of Epidemiology and Population Health, London School of Hygiene & Tropical Medicine, London, UK; 5 United Nations Educational, Scientific and Cultural Organization, Paris, France; 6 Malawi Epidemiology and Intervention Research Unit, Lilongwe, Malawi; 7 Institute of Health and Wellbeing, University of Glasgow, Glasgow, UK; 8 Centre for Fertility and Health, Norwegian Institute of Public Health, Oslo, Norway

**Keywords:** Nutrition, Paediatrics, Public health

## Abstract

The benefits of exclusive breastfeeding (EBF) for infant health and survival are well documented. However, its impact on educational outcomes has been contested and poorly researched in Africa. It has been hypothesised that positive associations reported in high-income countries can be attributed to residual confounding by socioeconomic status (SES). Our study investigated whether EBF duration in infancy is associated with educational attainment and age-for-grade attainment trajectories at school-age in rural Malawi. Longitudinal data on 1021 children at the Karonga demographic surveillance site in Malawi were analysed. Breastfeeding data were collected 3 months after birth and again at age one. The school grade of each child was recorded each year from age 6 until age 13. We calculated age-for-grade based on whether a child was at, over, or under the official expected age for a grade. Generalised estimating equations estimated the average effect of breastfeeding on age-for-grade. Latent class growth analysis identified age-for-grade trajectories, and multinomial logistic regression examined their associations with EBF. Maternal-child characteristics, SES, and HIV status were controlled. Overall, 35.9% of the children were exclusively breastfed for 6 months. Over-age for grade steadily increased from 9.6% at age 8 to 41.9% at age 13. There was some evidence that EBF for 6 months was associated with lower odds of being over-age for grade than EBF for less than 3 months (aOR = 0.82, 95%CI = 0.64–1.06). In subgroup analyses, children exclusively breastfed for 6 months in infancy were less likely to be over-age for grades between ages 6–9 (aOR = 0.64, 95%CI = 0.43–0.94). Latent class growth analysis also provided some evidence that EBF reduced the odds of falling behind in the early school grades (aOR = 0.66, 95%CI = 0.41–1.08) but not later. Our study adds to the growing evidence that EBF for 6 months has benefits beyond infant health and survival, supporting the WHO's recommendation on EBF.

## Introduction

Optimal breastfeeding has been linked to improved cognitive development and higher intelligence test scores in several studies. For example, in a systematic review and meta-analysis of studies mainly from high-income countries that controlled for home stimulation and measures of socioeconomic status, breastfed children scored 3.4 points higher on intelligence tests than non-breastfed children^1^. Not only has breastfeeding been linked to improved cognitive functions, but there is growing evidence of a positive association between breastfeeding and educational outcomes^2–5^. In high-income countries, the evidence supporting these positive associations has been relatively consistent over the last few decades. Heikkila et al.^2^, for example, found higher educational achievement among five-year-olds in the United Kingdom who were exclusively breastfed for a longer duration. In Australia, ten-year-olds who were predominantly breastfed for 6 months had higher academic scores than those who were breastfed for less than 6 months^4^. Among adolescents in the United States, being breastfed was associated with a higher grade point average compared to those not breastfed^5^.

While the exact mechanism is unclear, it has been proposed that certain components of human breastmilk, including human milk oligosaccharides, arachidonic acid, and docosahexaenoic acid, modulate the neurodevelopmental processes of the newborn brain in the first few months after birth, a time of rapid growth and maturation of the brain^6–9^. From this perspective, if breastfeeding truly has a biological (direct nutritional) effect on intelligence and educational outcomes, we should observe it across all populations and settings, not just in high-income countries. For instance, breastfed children have a reduced risk of childhood gastrointestinal and respiratory tract infections across populations regardless of country income level^10^.

Nevertheless, in low and middle-income countries (LMICs), the evidence on the effects of breastfeeding on intelligence and educational outcomes is unclear and mostly inconsistent with those from high-income countries^11,12^. In Brazil, for example, Victora et al.^13^*,* found that children breastfed for 12 months or more had 0.91 more years of education than those breastfed for less than 1 month. Among children aged 6–18 in India, breastfeeding for more than 12 months was associated with 0.12–0.19 more years of education than breastfeeding for less than 12 months^14^. However, no association was observed between breastfeeding and educational attainment when Horta et al.^15^*,* studied birth cohorts in the Philippines, Guatemala, India, and Brazil. Similarly, a recent study in Turkey found no evidence of an association between breastfeeding and academic performance^16^.

Some have questioned the consistent positive effects of breastfeeding on educational outcomes in studies conducted in high-income countries because of the conflicting results from low-income settings. It has been suggested that the positive effects observed in high-income countries may be attributable to residual confounding resulting from insufficient statistical control for socioeconomic status and not a direct effect of breastmilk^12,13,17,18^. This hypothesis is supported by the fact that in high-income settings, women with higher socioeconomic status breastfeed longer than those with lower socioeconomic status, and maternal education strongly correlates with offspring cognitive abilities and educational outcomes^13,19–21^.

Studies from sub-Saharan Africa are scarce in this decades-old debate. In a recent systematic review, we found that only two studies have investigated the effects of breastfeeding on educational attainment in sub-Saharan Africa^12^. Both studies were conducted in South Africa. One found weak evidence for an association between exclusive breastfeeding and grade repetition^22^, while the other study found no evidence of an association between breastfeeding and school attainment or completion^15^. However, generalising the South African findings to other African countries would be naive, given the varying associations reported in earlier studies from different settings. There are also important differences in childhood adversities (such as HIV exposure) and non-socioeconomic (sociocultural, political, and educational system-related) factors that affect educational attainment across sub-Saharan African countries^23^. In addition, previous studies, including those carried out in high-income settings, examined educational attainment at one point in time. It is possible that these studies might have missed a child's educational attainment at other time points because academic growth is not always linear. Using longitudinal birth cohort data from the Karonga Health and Demographic Surveillance System (HDSS) in Malawi, we examined primary school progression over eight years and assessed the effects of exclusive breastfeeding duration on educational attainment and age-for-grade attainment trajectories from age six when the children started primary school through to age 13.

## Methods

### Participants and data

We used prospective, longitudinal data from the Karonga HDSS in northern Malawi. The HDSS is situated in the southern part of the Karonga District and includes 42,000 individuals living in 7000 households^24,25^. Most of the population lives in rural areas, and a majority of them engage in subsistence farming, fishing, and trading. The HDSS undertakes continuous demographic surveillance in the area to collect socioeconomic, demographic, and household-level data and information on vital events and migration of individuals of all ages^26^. It started with a baseline census between 2002 and 2004. Births to women in the baseline survey were reported, and field staff enrolled the children as part of the demographic surveillance follow-up visits^27^. Data were collected on maternal and child characteristics, including child anthropometry, birth characteristics, breastfeeding and other feeding practices, and household socioeconomic status when the children were 0–3 months old. The birth cohort was revisited at age 12 months, and the feeding data and other infant and family characteristics were updated. Annual surveys and re-census were conducted in the HDSS to periodically update existing data and collect new information, including children's schooling, anthropometry, and vital events. For the present analysis, we restricted the study population to birth cohort members between the ages of six and 13 (primary school age in Malawi) with at least one data point on current primary school grade. In Malawi, school enrolment at public primary schools is free, and children do not have to pass an entrance examination to enrol in school^24^.

### Study measures

#### Outcome variable

**Figure 1:**
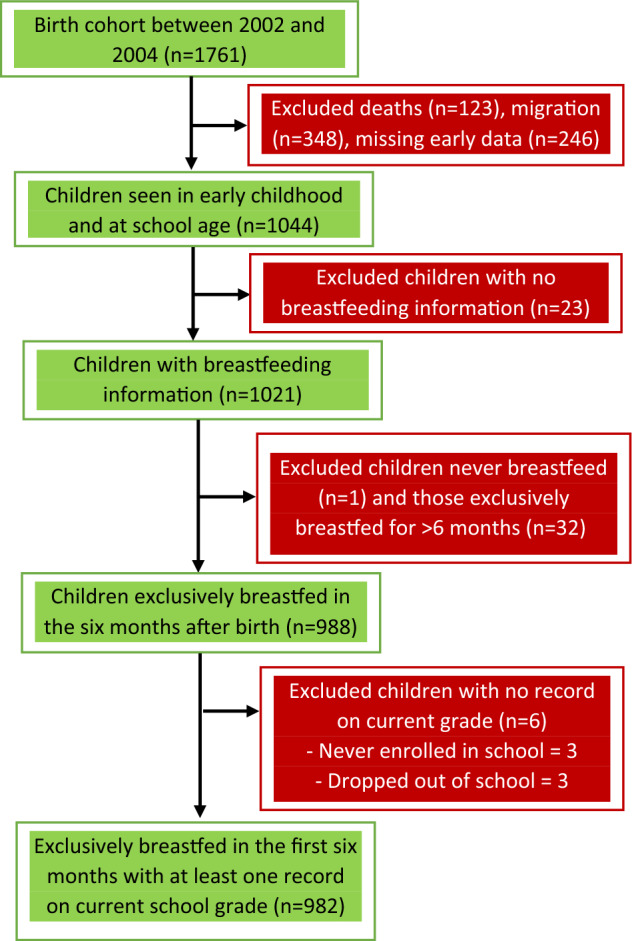
Flow of study subjects (The cohort was defined as in Sunny et al.^27^ which gives more detail on the exclusions).

The main outcome of this study was age-for-grade, defined as the age at which a child would be expected to enter a given grade if they had begun primary school on time and advanced without repeating or skipping a grade^28^. In this cohort, 96.3% (n = 953) of the children started primary school at age six or earlier, 3.4% (n = 34) started at age 7, and 0.3% (n = 3) started at age 8, so age-for-grade was primarily a measure of progress. Information on the current grade of each child in the cohort was collected at multiple time points starting in 2007, when they were about four years old and updated annually until 2015, when they were about thirteen years old. Public primary schools in Malawi consist of eight grades, and children are expected to enter grade 1 at age six and continue for eight years until they complete grade eight at age thirteen. For example, children are expected to enter grades 5, 6, and 7 at ages 10, 11, and 12, respectively. We calculated age-for-grade based on whether a child was at, over, or under the official expected age for entry into a grade for all the time points for which data were available for a given child. A child was considered under-age for grade if they were one or more years younger than the official age for the grade, on-time if they were of the official age or one year older than the official age for the grade, and over-age if they were two or more years older than the official age for the grade^24,29^. Over-age for grade is a sign that a child is struggling academically, leading them to repeat grades. Six children did not have data on age-for-grade and were excluded from further analysis (Fig. 1). Three of the six children with no current grade record were never enrolled, and three left school (one each at the ages of 5, 6, and 11), and their records were not taken.

#### Exposure variable

Exclusive breastfeeding in the first 6 months after birth is the main exposure. Mothers reported information on breastfeeding and other infant feeding practices at baseline when the children were 0–3 months old and at follow-up when the average age of children was 12 months (SD = 0.03 months). Mothers were asked if the child was breastfeeding and how old the child was when other kinds of milk, formula, water, solid foods, and other foods were introduced. We defined exclusive breastfeeding as feeding a child with only breastmilk in the first 6 months after birth. The duration of exclusive breastfeeding was categorised into 0–2 months, 3–5 months, and 6 months. We excluded from the analysis the few children (n = 32, 3.1%) who were exclusively breastfed for longer than the recommended 6 months.

#### Potential confounders and moderators

Potential confounding variables were identified based on prior research^5,12,30^ and included child sex, household wealth at birth, age of mother at birth, birth order of child, maternal HIV status, maternal education, paternal education, maternal occupation, paternal occupation, and distance to a tarmac road. The estimation of household wealth at birth was based on a principal component analysis of household items, dwelling characteristics, ownership of consumer durables, and utility access, as described in an earlier publication^27^. In addition, we decided a priori to assess whether the association between breastfeeding and educational attainment varied by sex and age at school assessment (6–9 and 10–13 years).

### Data analysis

We estimated the number of children exclusively breastfed for 0–2 months, 3–5 months, and 6 months to determine the prevalence of exclusive breastfeeding. The characteristics of the children were summarised and stratified by the exclusive breastfeeding groups. Age-for-grade was recoded as a binary response variable with 1 = over-age for grade and 0 = underage/on-time for grade. Three sets of analyses were performed to determine the association between exclusive breastfeeding and age-for-grade.

First, binary logistic regression was used to model the association between exclusive breastfeeding and being over-age for grade using one observation per child and restricting the analysis to schooling assessments at the point closest to age 11.5 years (mid-point between 10 and 13), as most children had information on current grade at these ages. However, this approach does not make use of the longitudinal nature of the data and repeated schooling assessment.

In the second approach, we used generalised estimation equations (GEE)^31^ with an exchangeable correlation structure to estimate the effect of exclusive breastfeeding duration on age-for-grade measured at multiple time points between ages 6 and 13, taking into account the lack of independence between repeated school grade measurements for each child. In addition, the GEE approach can handle unbalanced data (uneven number of assessments per individual) and generate unbiased estimates even if the within-cluster correlation structure is misspecified^32,33^. We fitted separate GEE models for children aged 6–9 and 10–13 and for girls and boys because we hypothesised that the effect of exclusive breastfeeding on age-for-grade is likely to differ by age and sex^5,12^.

However, since the GEE approach estimates the average population effects of exposure on the outcome, this can mask important differences between hidden subgroups of the study population since academic growth can be nonlinear. For example, some children may perform poorly in early grades but catch up in the later grades, while others who did well in the early grades may do poorly in later grades. When such heterogeneity is ignored, results may be biased^34^.

Hence, the third approach used Latent Class Growth Analysis (LCGA)^35^ to identify unobserved homogenous subgroups (latent classes) within the study population that shared common trajectories of grade progression from age 6–13 using the “traj” macro in Stata^36^. LCGA is a person-centred technique that uses repeated measures of a single outcome variable across age or time to delineate a latent class model in which the classes represent different trajectories for the outcome variable^37,38^. The underlying assumption of LCGA is that individuals designated to the same latent class follow the same pattern of change over time^39,40^.

Unconditional models with one through to five latent classes were tested through an iterative process using a combination of linear, quadratic, and cubic polynomial functions (trajectory shape) in a logit model for the longitudinal binary outcome, age-for-grade. Models with more than five classes produced anomalous growth curves, and the model fit statistics started to deteriorate and, in most cases, failed to converge. Age at school assessment was the indicator of time. We included only children with at least three school assessments in the LCGA. The maximum probability assignment rule^41^ was used to assign participants to the latent class they had the highest posterior membership probability.

To determine the optimal number of latent classes, we compared the Bayesian information criterion [BIC] and Akaike Information Criterion [AIC]) values of the fitted latent class models. Lower BIC and AIC values indicate a balance between good model fit and parsimony^40,42–45^. However, because they are highly sensitive to sample size, researchers are advised to use BIC and AIC values in conjunction with other measures of model adequacy^42–44^. Thus, in addition to the information criteria, we utilised a number of model adequacy diagnostics, including the average posterior probability (AvePP) of class assignment, entropy (a measure of the accuracy of class classification), class size, and the practical interpretability (meaningfulness) of the latent class trajectories. Values of entropy near 1 (minimum 0.5), AvePP ≥ 0.70, and at least 5% of participants in each class have been proposed as indicators of high assignment accuracy though there are no conventional cut-offs^40,42–45^. We plotted individual-level grade progressions in each class of the latent class models to examine the meaningfulness and plausibility of the trajectories and how well they captured individual-level age-for-grade patterns. Because there are no definitive decision criteria, it is suggested that the selection of the optimal model balances between meaningful trajectories and model adequacy^35,40,42,43^. We used the approach described to model separate latent class trajectories for girls and boys because of differences in their academic progression^46^.

**Figure 2:**
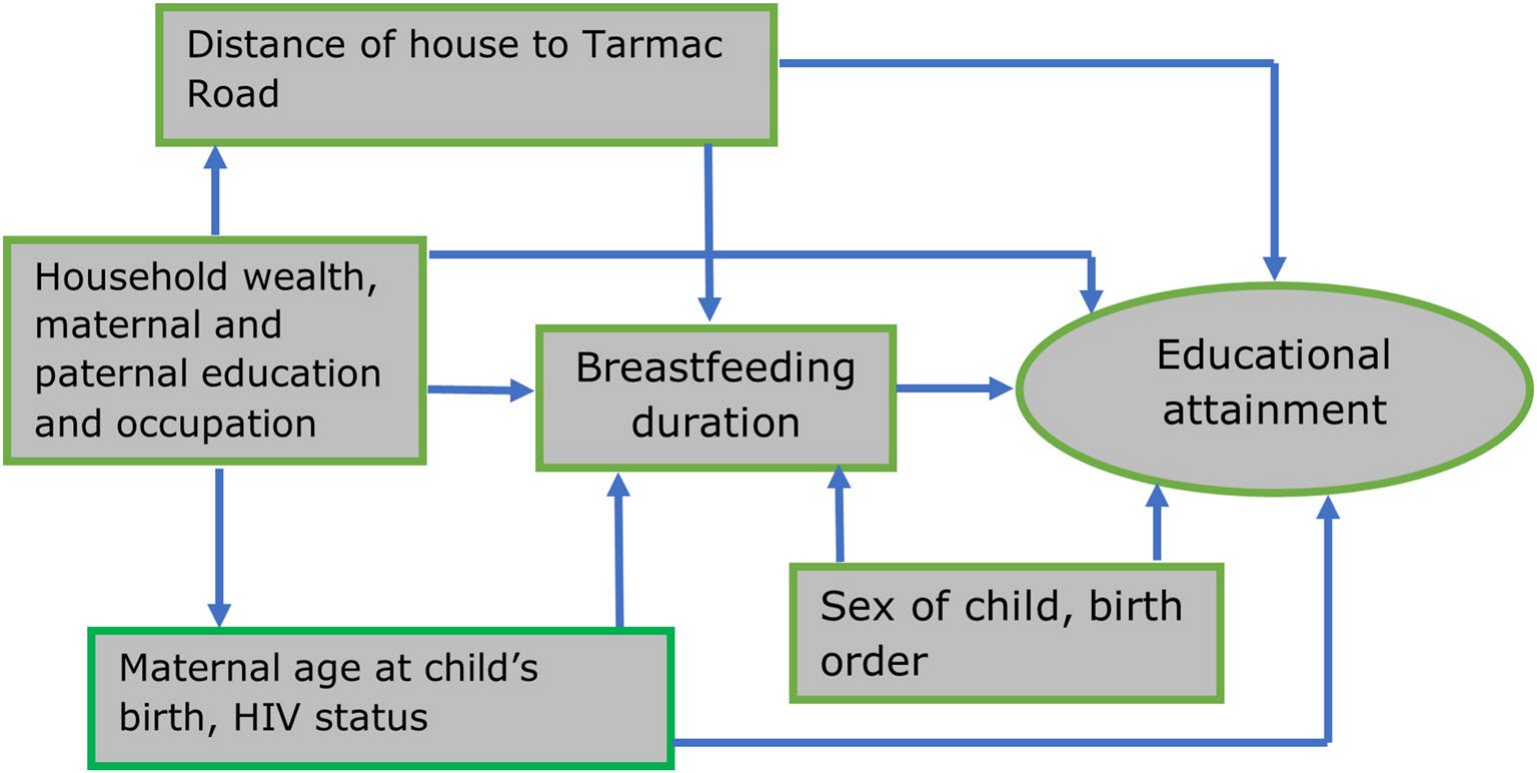
Directed acyclic graph showing the relationship between the exposure, outcome, and confounding variables.

The participants probabilities of belonging to each trajectory class were used as a categorical variable in a multivariable multinomial logistic regression model to determine the association between exclusive breastfeeding duration and the latent age-for-grade trajectories. We used the trajectory with better grade progression as the reference group. In all the analyses, we adjusted for the baseline confounders outlined in the directed acyclic graph in Fig. 2, with these covariates included as categorical variables using the groupings presented in Table 1. All analyses were performed using Stata version 17.

**Table 1:** Distribution of exclusive breastfeeding duration according to sociodemographic characteristics of the study population.

	Total (n = 1021)	Children exclusively breastfed in first 6 months (n = 988)^a^	Duration of exclusive breastfeeding		
			0–2 months	3–5 months	6 months
	n (%)	n (%)	n (%)	n (%)	n (%)
All			181 (18.3)	452 (45.8)	355 (35.9)
Sex of child					
Female	491 (48.1)	477 (48.3)	80 (16.8)	219 (45.9)	178 (37.3)
Male	530 (51.9)	511 (51.7)	101 (19.8)	233 (45.6)	177 (34.6)
Maternal HIV status					
Negative	893 (87.5)	867 (87.8)	167 (19.3)	396 (45.7)	304 (35.0)
Positive	28 (2.7)	27 (2.7)	3 (11.1)	11 (40.7)	13 (48.2)
Unknown	100 (9.8)	94 (9.5)	11 (11.7)	45 (47.9)	38 (40.4)
Mother education					
None/incomplete primary	493 (48.3)	476 (48.3)	97 (20.4)	219 (45.0)	160 (33.6)
Complete primary	320 (31.3)	308 (31.3)	57 (18.5)	138 (44.8)	113 (36.7)
Any secondary	205 (20.1)	201 (20.4)	27 (13.4)	94 (46.8)	80 (39.8)
Missing	3 (0.3)	–			
Father education					
None/incomplete primary	233 (22.8)	224 (23.9)	48 (21.4)	114 (50.9)	62 (27.7)
Complete primary	342 (33.5)	333 (35.5)	58 (17.4)	147 (44.1)	128 (38.4)
Any secondary	395 (38.7)	381 (40.6)	66 (17.3)	168 (44.1)	147 (38.6)
Missing	51 (5.0)	–			
Mother occupation					
Not working	207 (20.3)	200 (20.3)	36 (18.0)	78 (39.0)	86 (43.0)
Subsistence farmer	792 (77.5)	768 (78.0)	145 (18.9)	364 (47.4)	259 (33.7)
Others	19(1.9)	17 (1.7)	0 (0.0)	8 (47.1)	9 (52.9)
Missing	3 (0.3)	–			
Father occupation					
Not working	52 (5.1)	50 (5.3)	10 (20.0)	21 (42.0)	19 (38.0)
Subsistence farmer	601 (58.9)	587 (62.8)	121 (20.6)	274 (46.7)	192 (32.7)
Others	313 (30.6)	298 (31.9)	41 (13.8)	133 (44.6)	124 (41.6)
Missing	55 (5.4)	–			
Household wealth at birth					
Poorest	349 (34.2)	339 (35.3)	72 (21.2)	157 (46.3)	110 (32.5)
Middle	336 (32.9)	324 (33.8)	65 (20.1)	138 (42.6)	121 (37.4)
Least poor	307 (30.1)	296 (30.9)	39 (13.2)	142 (48.0)	115 (38.8)
Missing	29 (2.8)	–			
Age of mother at birth					
< 20	209 (20.4)	203 (20.5)	45 (22.2)	89 (43.8)	69 (34.0)
20–30	600 (58.8)	583 (59.0)	99 (17.0)	278 (47.7)	206 (35.3)
> 30	212 (20.8)	202 (20.5)	37 (18.3)	85 (42.1)	80 (39.6)
Birth order of child					
1	227 (22.2)	223 (23.4)	55 (24.7)	101 (45.3)	67 (30.0)
2–3	332 (32.5)	321 (33.7)	48 (15.0)	158 (49.2)	115 (35.8)
4 or more	424 (41.5)	409 (42.9)	73 (17.9)	176 (43.0)	160 (39.1)
Missing	38 (3.7)	–			
Distance to tarmac road					
Within 1 km	513 (50.2)	493 (49.9)	78 (15.8)	224 (45.5)	191 (38.7)
More than 1 km	508 (49.8)	495 (50.1)	103 (20.8)	228 (46.1)	164 (33.1)

^a^Excludes children who were exclusively breastfed for more than 6 months (n = 32).

### Missing data

The amount of data missing at baseline was low, ranging from 0.3% (maternal education and maternal occupation) to 5.4% (paternal occupation) (see online Supplementary Table 1). Missing data mechanisms of baseline confounding variables were investigated and assumed to be missing at random. We performed multiple imputations by chained equations (MICE) in Stata to minimise bias and loss of precision and power from discarding observations with missing values^47^. Thirty imputed datasets were generated under fifteen iterations for each imputation model. All variables in the substantive analyses were included in the imputation models to predict the distribution of the missing values. However, only six of the ten baseline confounding variables with missing data were imputed (maternal education, maternal occupation, paternal education, paternal occupation, household wealth, and birth order). We did not impute missing values in the outcome variable. The substantive analyses described above were conducted on the imputed dataset, and the estimates combined and standard errors estimated using Rubin's rules^48^. For the multinomial logistic regression analysis, separate MICE models were fitted for the entire sample as well as for boys and girls.

In sensitivity analysis, we repeated the binary logistic regression, multinomial logistic regression, and GEE analyses using only individuals with complete data to assess the reliability of our substantive analysis that used imputed data.

### Ethical consideration

The National Health Sciences Research Committee in Malawi (protocol number #1072) and the Research Ethics Committee of the London School of Hygiene and Tropical Medicine (protocol number #6303) granted ethical approval for the primary study. Permission and informed consent were sought from traditional leaders and village headmen, and verbal informed consent for participation was obtained from each household head and participating household members (parents provided informed consent for their children's participation) after the purpose of the surveillance were explained. Those who refused to give consent were not interviewed^25,27^. Ethics approval for the present analysis was granted by the research ethics committee of the London School of Hygiene and Tropical Medicine (Ethics Ref: 26468). In addition, we confirm that the analysis was performed in accordance with relevant guidelines/regulations, and in accordance with the Declaration of Helsinki.

## Results

### Characteristics of the study sample

There were 1761 births to women in the HDSS between 2002 and 2004, and 1044 were successfully followed up for at least 4 years (Fig. 1). Loss to follow-up was largely due to migration, deaths, and missing early data. 1021 children had information about breastfeeding practices and were prospectively followed for six to thirteen years. Descriptive statistics in Table 1 show that slightly more than half (51.9%) of the children were male, and half (50.2%) lived within 1 km of a tarmac road. There were 227 (23.1%) first-order births, and 58.8% of the mothers were between the ages of 20 and 30 when the children in the cohort were born. While 40.7% of the children’s fathers had at least some secondary education, only 20.2% of their mothers had the same level of education. Both parents were largely subsistence farmers. The majority (87.5%) of the mothers were HIV-negative, and only 2.7% were known to be HIV-positive. Almost all the children were breastfed (99.9%).

### Duration of exclusive breastfeeding

Most (45.8%) children were exclusively breastfed for 3–5 months (Table 1). Only 35.9% were exclusively breastfed for the recommended 6 months. There was no sex difference in the prevalence of exclusive breastfeeding and no significant association between maternal education and duration of exclusive breastfeeding. However, the prevalence of exclusive breastfeeding for 6 months was higher with increasing household wealth and paternal education. Mothers who did not work exclusively breastfed for a longer duration than those who were subsistence farmers. Similarly, children whose fathers were unemployed were more likely to be exclusively breastfed for 6 months than those whose fathers were subsistence farmers. The order of births was associated with exclusive breastfeeding, with a greater percentage of children of higher birth order more likely to be exclusively breastfed for 6 months than those of lower birth order.

### Age-for-grade

**Figure 3:**
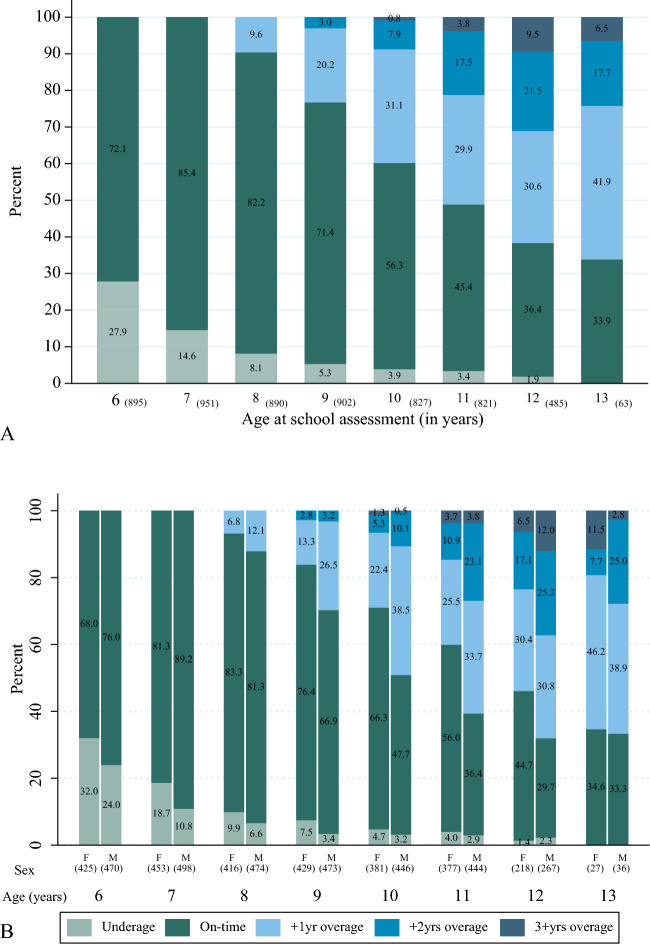
Distribution of age-for-grade by (**A**) age and (**B**) age and sex [F = female M = male]. Note: underage = one or more years younger than the official grade age; on time = at the official grade age; +1yr overage = one year older than the official grade age; +2yrs overage = two years older than the official grade age; and 3+yrs overage = three or more years older than the official grade age. Numbers in parentheses represent the number (n) of children in each group.

Of the eight possible time points at which age-for-grade was assessed, 1.9% had all eight recorded, 24.1% had 7, 43.3% had 6, and 13.8% had 5, and the remainder had between one and four school assessments. At age six, data on age-for-grade was collected for 895 children. The number of children increased to 951 at age seven but subsequently decreased to 902 at age nine, 821 at age 11, 485 at age 12, and 63 at age 13 (Fig. 3A). The earliest children began to fall behind in school was age eight, and the proportion of children under-age or on time for grades steadily decreased. By age 13, no child was underage-for-grade, and 41.9% of the children were 1 year older than their grade level. A similar upward trend was observed until age twelve for children who were 2–3 years over-age for their grades. Figure 3B shows that more boys than girls were assessed at each age of schooling. However, girls were more likely than boys to be underage and on-time for a grade.

### Association between exclusive breastfeeding duration and age-for-grade at age 11.5

Using binary logistic regression, we found no evidence of an association between exclusive breastfeeding duration and age-for-grade between ages 10–13 in the overall sample and sex-stratified analyses (Table 2). Results based on complete case analysis excluding participants with missing data were consistent with this finding (Supplementary Table 5).

**Table 2:** Binary logistic regression analysis of the association between exclusive breastfeeding duration and age-for-grade attainment at age 11.5 (mid-point of age 10–13) in Malawi (n = 887).

	N	Age-for-grade		Unadjusted odds ratio (95% CI)	P-value	Adjusted odds ratio (95% CI)	P-value
		Underage or on-time for grade	Overage for grade				
		n (%)	n (%)				
Model 1 age 6–13 Both sexes: duration of exclusive breastfeeding (n = 887)							
0–2 months	161	73 (45.3)	88 (54.7)	1.00	0.36	1.00	0.85
3–5 months	409	208 (50.9)	201 (49.1)	0.80 (0.56–1.16)		0.92 (0.62–1.38)	
6 months	317	165 (52.1)	152 (47.9)	0.76 (0.52–1.12)		1.01 (0.66–1.53)	
Model 2 Girls, Age 6–13: duration of exclusive breastfeeding (n = 417)							
0–2 months	69	40 (58.0)	29 (42.0)	1.00	0.85	1.00	0.46
3–5 months	196	121 (61.7)	75 (38.3)	0.85 (0.49–1.49)		0.86 (0.47–1.58)	
6 months	152	91 (59.9)	61 (40.1)	0.92 (0.52–1.65)		1.16 (0.62–2.18)	
Model 3 Boys, Age 6–13: duration of exclusive breastfeeding (n = 470)							
0–2 months	92	33 (35.9)	59 (64.1)	1.00	0.37	1.00	0.85
3–5 months	213	87 (40.9)	126 (59.1)	0.81 (0.49–1.34)		1.04 (0.60–1.80)	
6 months	165	74 (44.8)	91 (55.2)	0.69 (0.41–1.16)		0.92 (0.52–1.62)	

Note: We controlled for child’s sex, household wealth at birth, age of mother at birth, birth order of child, maternal HIV status, mother education, father education, mother occupation, father occupation, and distance to a tarmac road in the adjusted analysis. MICE was used to impute missing values in household wealth (n = 29), birth order (n = 38), mother education (n = 3), father education (n = 51), father occupation (n = 55), and mother occupation (n = 3).

### Association between exclusive breastfeeding duration and repeated measures of age-for-grade attainment from age 6 through to 13

In the generalised estimations equation analysis (Table 3), adjusted results provided some evidence that children exclusively breastfed for 6 months were less likely to be over-age for grade than those exclusively breastfed for less than 3 months (aOR = 0.82; 95%CI = 0.64–1.06). When we stratified the analysis by age, results from the adjusted analysis showed evidence of a dose–response effect of exclusive breastfeeding duration on age-for-grade between ages 6 and 9. Children exclusively breastfed for 6 months had 36% lower odds of being over-age for grades between ages six and nine than those exclusively breastfed for less than 3 months (aOR = 0.64; 95%CI = 0.43–0.94). There was no evidence of an association between exclusive breastfeeding duration and age-for-grade between ages 10 and 13. In the sex-stratified analysis, the adjusted results showed no evidence of an association between exclusive breastfeeding and age-for-grade. Complete case analysis excluding participants with missing data produced consistent results (Supplementary Table 6).

**Table 3:** Generalised estimating equations analysis of the effects of exclusive breastfeeding duration on age-for-grade attainment among children aged 6–13 in Malawi (N = 982).

	Unadjusted odds ratio (95% CI)	P-value	Adjusted odds ratio (95% CI)	P-value
Model 1 Age 6–13 Both sexes: Duration of exclusive breastfeeding (n = 982)				
0–2 months	1.00	0.003*	1.00	0.13*
3–5 months	0.81 (0.63–1.04)		0.90 (0.70–1.14)	
6 months	0.68 (0.52–0.88)		0.82 (0.64–1.06)	
Model 2 Age 6–9 Both sexes: Duration of exclusive breastfeeding (n = 972)				
0–2 months	1.00	< 0.001*	1.00	0.02*
3–5 months	0.75 (0.53–1.08)		0.87 (0.61–1.24)	
6 months	0.50 (0.34–0.74)		0.64 (0.43–0.94)	
Model 3 Age 10–13 Both sexes: Duration of exclusive breastfeeding (n = 891)				
0–2 months	1.00	0.19	1.00	0.88
3–5 months	0.80 (0.57–1.12)		0.91 (0.64–1.30)	
6 months	0.72 (0.50–1.03)		0.93 (0.64–1.35)	
Model 4 Girls, Age 6–13: Duration of exclusive breastfeeding (n = 475)				
0–2 months	1.00	0.18	1.00	0.56
3–5 months	0.92 (0.60–1.40)		0.91 (0.59–1.39)	
6 months	0.71 (0.46–1.09)		0.80 (0.52–1.24)	
Model 5 Boys, Age 6–13: Duration of exclusive breastfeeding (n = 508)				
0–2 months	1.00	0.08	1.00	0.50
3–5 months	0.77 (0.57–1.05)		0.91 (0.68–1.22)	
6 months	0.69 (0.49–0.95)		0.83 (0.61–1.13)	

Note: We controlled for child’s sex, household wealth at birth, age of mother at birth, birth order of child, maternal HIV status, mother education, father education, mother occupation, father occupation, and distance to a tarmac road in the adjusted analysis. MICE was used to impute missing values in household wealth (n = 29), birth order (n = 38), mother education (n = 3), father education (n = 51), father occupation (n = 55), and mother occupation (n = 3).

*Test for linear trend.

### Latent class growth trajectories and patterns of grade progression

The few children with less than three school grade records were excluded from LCGA (n = 63). Following the evaluation of the goodness of fit statistics and model adequacy diagnostics for the fitted latent classes (Supplementary Table 2), the three-class and four-class models were considered suitable candidates to appropriately capture the grade progression of the study sample. These models both had more than 20% of participants in each class and entropy values within the acceptable range (three-class model = 0.82; four-class model = 0.64). The four-class model had the lowest BIC (− 1544.69) and AIC (− 1515.54) values, whereas the classes in the three-class model had the highest AvePP of assignment values and odds of correct classification. However, the trajectories of the four-class model best represented individual-level grade progression, demonstrated acceptable class separation, and were practically meaningful (Supplementary Fig. 1). Based on these assessments, the four-class model was selected as the optimal model.

**Figure 4:**
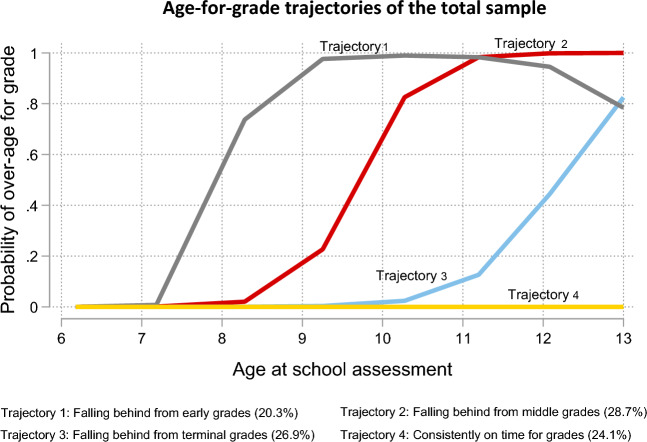
Age-for-grade attainment trajectories based on latent class growth modelling of schooling data from age 6 to 13 (Early grades = grades 1–3, middle grades = grades 4–6 and terminal grades = grades 7–8).

Each trajectory was named based on the shape of the curve and the age at which the children in that trajectory started to fall behind in school (Fig. 4). Trajectory 1 represented children over-age for grade in the early grades of primary school, starting from around age 8 (falling behind from early grades). Children in trajectory 2 were over-age for grades in middle primary school when they were about 9–10 years (falling behind from middle grades). Those in trajectory 3 became over-age for grades at the terminal stages of primary school when they were about 11–12 (falling behind from terminal grades). Trajectory 4 represents children on time for all the grades from age six to thirteen (consistently on time for grades).

**Figure 5:**
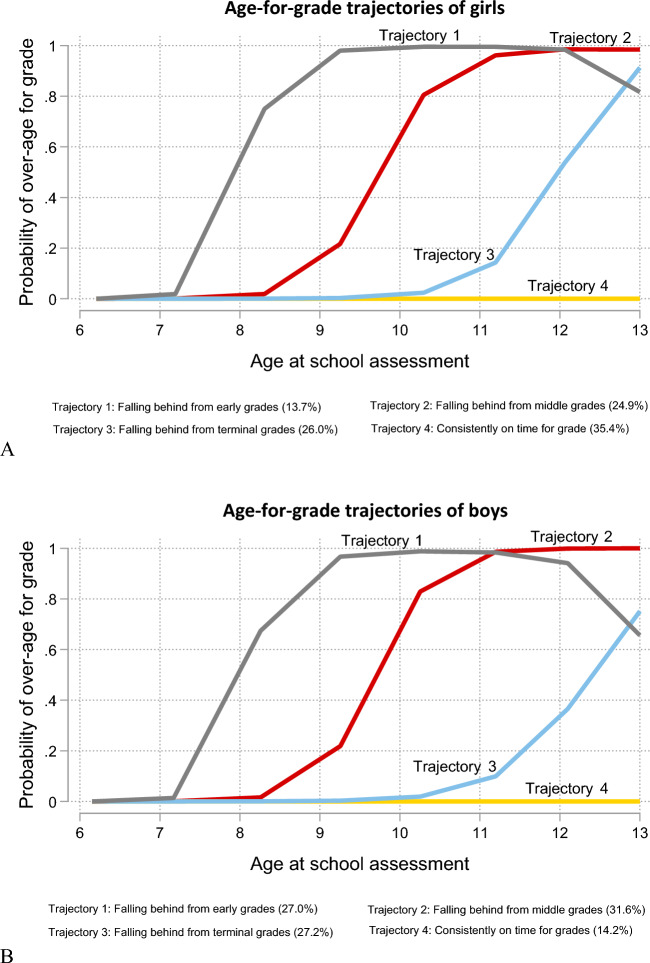
Age-for-grade attainment trajectories of (**A**) Girls and (**B**) Boys based on latent class growth modelling of schooling data from age 6 to 13 (Early grades = grades 1–3, middle grades = grades 4–6 and terminal grades = grades 7–8).

We evaluated the sex-stratified latent class models using the approach described (Supplementary Tables 3, 4, Supplementary Figs. 2 and 3) and found that a four-class model was optimal for both girls and boys (Fig. 5A and B).

Table 4 shows the distribution of the overall age-for-grade trajectories across the participants’ sociodemographic characteristics. Overall, 24.1% of the children progressed without falling behind; 26.9% started falling behind in the terminal grades, and 28.7% and 20.3% started falling behind in the middle and early grades, respectively. A greater percentage of the children who fell behind in early grades were boys. The percentage of children consistently on time for grades increased with increasing paternal education and household wealth. Being consistently on time for grade was more common among later-order births and children living within 1 km of a tarmac road.

**Table 4:** Distribution of the overall age-for-grade trajectories of children with three or more repeated school assessments according to sociodemographic characteristics of the study sample (n = 952).

	Categories	Age-for-grade trajectories			
		Trajectory 1	Trajectory 2	Trajectory 3	Trajectory 4
		Falling behind from early grades	Falling behind from middle grades	Falling behind from terminal grades	Consistently on time for grade
		n (%)	n (%)	n (%)	n (%)
Overall	Age-for-grade trajectories	193 (20.3)	273 (28.7)	256 (26.9)	230 (24.1)
Duration of exclusive breastfeeding	0–2 months	44 (23.6)	46 (17.5)	40 (16.0)	36 (16.2)
	3–5 months	89 (48.1)	119 (44.8)	119 (48.0)	97 (43.6)
	6 months	53 (28.3)	100 (37.7)	89 (36.0)	89 (40.1)
Sex of child	Female	65 (33.5)	113 (41.4)	142 (55.4)	132 (57.6)
	Male	128 (66.5)	160 (58.6)	114 (44.6)	98 (42.4)
Maternal HIV status	Negative	172 (89.1)	251 (91.9)	224 (87.4)	198 (86.3)
	Positive	3 (1.6)	6 (2.2)	7 (2.9)	8 (3.3)
	Missing	18 (9.3)	16 (5.9)	25 (9.7)	24 (10.4)
Mother education	None/incomplete primary	127 (65.8)	149 (55.1)	104 (40.7)	87 (38.1)
	Complete primary	44 (22.6)	82 (30.2)	91 (35.8)	83 (36.2)
	Any secondary	22 (11.6)	40 (14.7)	61 (23.5)	59 (25.7)
Father education	None/incomplete primary	68 (37.2)	76 (29.1)	40 (16.6)	30 (14.0)
	Complete primary	68 (37.2)	97 (37.0)	87 (35.6)	74 (34.4)
	Any secondary	47 (25.6)	89 (33.9)	116 (47.8)	111 (51.6)
Mother occupation	Not working	33 (17.2)	38 (14.0)	58 (22.7)	52 (22.6)
	Subsistence farmer	159 (82.2)	230 (84.8)	190 (74.3)	171 (74.5)
	Others	1 (0.6)	3 (1.2)	8 (3.0)	7 (2.9)
Father occupation	Not working	9 (5.1)	12 (4.6)	15 (6.3)	13 (6.3)
	Subsistence farmer	131 (72.0)	169 (58.6)	142 (58.6)	119 (55.6)
	Others	42 (22.9)	80 (30.8)	85 (35.1)	82 (38.1)
Household wealth at birth	Poorest	87 (47.0)	112 (42.1)	71 (28.5)	54 (24.4)
	Middle	62 (33.5)	91 (34.3)	85 (34.0)	75 (33.6)
	Least poor	36 (19.5)	62 (23.6)	93 (37.5)	94 (42.0)
Age of mother at birth	< 20	47 (24.6)	50 (18.3)	53 (20.9)	45 (19.7)
	20–30	102 (52.8)	165 (60.6)	149 (58.2)	134 (58.2)
	> 30	44 (22.7)	57 (21.1)	54 (20.9)	51 (22.1)
Birth order of child	1	47 (25.4)	57 (21.6)	56 (22.5)	48 (21.9)
	2–3	52 (28.3)	88 (33.6)	90 (36.2)	79 (35.6)
	4 or more	85 (46.3)	118 (44.9)	102 (41.3)	94 (42.5)
Distance to tarmac road	Within 1 km	68 (35.2)	132 (48.4)	143 (55.8)	128 (55.7)
	More than 1 km	125 (64.8)	141 (51.6)	113 (44.2)	102 (44.3)

Note: Early grades = grades 1–3, middle grades = grades 4–6 and terminal grades = grades 7–8.

### Association between exclusive breastfeeding duration and age-for-grade trajectories identified through latent class growth modelling

Compared to being consistently on time for grades, children exclusively breastfed for 6 months were less likely to fall behind in early grades than those exclusively breastfed for less than 3 months in the unadjusted (OR = 0.48; 95%CI = 0.30–0.77) and adjusted (aOR = 0.66; 95%CI = 0.41–1.08) analyses (Table 5). There was no association between exclusive breastfeeding and later age-for-grade trajectories. In the sex-stratified analysis, there was no association between exclusive breastfeeding and age-for-grade trajectories in both the girls and boys subgroups. However, the pattern of the odds ratios was more apparent and consistent for boys than it was for girls. Complete case analysis excluding participants with missing data produced similar results (Supplementary Table 7).

**Table 5:** Multinomial logistic regression analysis of the association between exclusive breastfeeding duration and age-for-grade trajectories among children aged six to thirteen in Malawi.

	Falling behind from early grades vs consistently on time for grade		Falling behind from middle grades vs consistently on time for grade		Falling behind in terminal grades vs consistently on time for grade	
	OR (95% CI)	aOR (95% CI)	OR (95% CI)	aOR (95% CI)	OR (95% CI)	aOR (95% CI)
Model 1 age 6–13 Both sexes: duration of exclusive breastfeeding (n = 921)						
	P = 0.001	P = 0.07	P = 0.47	P = 0.68	P = 0.12	P = 0.20
0–2 months	1.00	1.00	1.00	1.00	1.00	1.00
3–5 months	0.76 (0.46–1.17)	0.90 (0.57–1.43)	0.95 (0.63–1.43)	1.06 (0.69–1.62)	1.11 (0.90–1.38)	1.13 (0.90–1.41)
6 months	0.48 (0.30–0.77)	0.66 (0.41–1.08)	0.87 (0.57–1.32)	1.10 (0.71–1.71)	0.91 (0.74–1.12)	0.94 (0.76–1.16)
Model 2 Girls, Age 6–13: duration of exclusive breastfeeding (n = 438)						
	P = 0.07	P = 0.25	P = 0.72	P = 0.35	P = 0.65	P = 0.79
0–2 months	1.00	1.00	1.00	1.00	1.00	1.00
3–5 months	0.99 (0.49–2.02)	1.01 (0.46–2.20)	1.01 (0.55–1.85)	0.99 (0.52–1.89)	1.24 (0.89–1.73)	1.25 (0.88–1.76)
6 months	0.56 (0.26–1.19)	0.67 (0.30–1.48)	1.10 (0.60–2.01)	1.28 (0.67–2.43)	1.02 (0.73–1.40)	1.04 (0.75–1.44)
Model 3 Boys, Age 6–13: duration of exclusive breastfeeding (n = 483)						
	P = 0.006	P = 0.07	P = 0.21	P = 0.67	P = 0.07	P = 0.09
0–2 months	1.00	1.00	1.00	1.00	1.00	1.00
3–5 months	0.65 (0.36–1.19)	0.87 (0.47–1.60)	0.91 (0.51–1.62)	1.13 (0.61–2.09)	1.00 (0.75–1.32)	1.01 (0.76–1.35)
6 months	0.42 (0.22–0.79)	0.57 (0.30–1.10)	0.71 (0.39–1.29)	0.93 (0.49–1.78)	0.81 (0.62–1.07)	0.83 (0.63–1.10)

P-values are from the test for linear trend.

Note: We controlled for child’s sex, household wealth at birth, age of mother at birth, birth order of child, maternal HIV status, mother education, father education, mother occupation, father occupation, and distance to a tarmac road in the adjusted analysis. MICE was used to impute missing values in household wealth (n = 29), birth order (n = 38), mother education (n = 3), father education (n = 51), father occupation (n = 55), and mother occupation (n = 3).

## Discussion

We used prospective, longitudinal data to examine whether exclusive breastfeeding duration in the first 6 months after birth is associated with educational attainment and age-for-grade trajectories between ages 6 and 13. We saw some evidence of a reduced likelihood of over-age for grade in children exclusively breastfed for 6 months compared to those exclusively breastfed for less than 3 months. Latent class growth analysis indicated that the effect was on those falling behind in the early grades of primary school rather than those who fell behind later. While the width of the confidence intervals and modest effects should be interpreted with caution, they are similar to the association found in the saving brains cohort in KwaZulu-Natal, South Africa, where researchers investigated the effects of exclusive breastfeeding on grade repetition in children aged 7–11 years^22^. Additionally, in a prespecified age subgroup analysis, we found evidence of a dose–response relationship between exclusive breastfeeding and educational attainment. Children exclusively breastfed for a longer duration in infancy were less likely to be over-age for grade at 6–9 years.

Our finding on the effects of exclusive breastfeeding on age-for-grade trajectories is novel, as no previous study has investigated breastfeeding’s effect on longitudinal trajectories of grade progression among homogenous subgroups. The majority of studies, including those that assessed educational attainment at multiple time points, used methods that assumed study participants progressed at the same level and estimated the average effect of breastfeeding on attainment, ignoring the nuances in progression in the population. The use of various analytical methods adds to the robustness of our findings. Unlike previous studies, our study population was almost entirely breastfed, and maternal education was not associated with the duration of exclusive breastfeeding. Even though adjustment for confounders, including socioeconomic status, generally attenuated the association between exclusive breastfeeding and age-for-grade in some subgroups, our ORs were consistent with reduced odds of falling behind in early school grades with longer duration of exclusive breastfeeding.

Our results are consistent with several earlier studies in varying settings^2,4,13,14,49^. Richards et al.^50^, for example, found a dose–response relationship between breastfeeding and higher educational attainment in the 1946 British birth cohort. In Brazil, longer breastfeeding duration was linked to more years of schooling completed at age 18 than breastfeeding for less than 1 month in a population with no socioeconomic patterning of breastfeeding^49^. Using data on sibling pairs from the National Longitudinal Study of Adolescent Health in the United States, Rees and Sabia found that breastfeeding was associated with a higher grade point average and a higher likelihood of graduating from high school and enrolling in college^51^.

The mechanisms underlying the improved educational attainment of optimally breastfed children have yet to be confirmed by research. However, one of the primary ways that breastfeeding may improve academic performance is through its role in brain development. Breastmilk contains a variety of nutrients and bioactive compounds necessary for brain development and cognitive function^52–55^. Breastmilk, for example, contains a high concentration of long-chain polyunsaturated fatty acids (LCPUFAs), which are required for brain and nervous system development^52,53^. Docosahexaenoic acid (DHA), one of the LCPUFAs in breastmilk, plays a crucial role in developing neural connections essential for learning and cognition^52,53,56^. Indeed, brain images of children and adolescents revealed that breastfed children have greater cortical thickness and significant increases in white and grey matter volume in many brain regions compared to non-breastfed children^57–61^. In addition to its direct nutritional benefits, breastfeeding promotes mother–child attachment and bonding^62^, which have been linked to enhanced cognitive and socioemotional development^63^, both of which are essential for academic success. However, studies have found positive effects of breastfeeding on cognitive development even after controlling for home stimulation^3,21^.

Our findings confirmed heterogeneity in grade progression across primary school. To promote good primary school progression, educational interventions should consider the trajectories identified in this study, noting when children begin to fall behind. Interventions should prioritise children whose parents are illiterate, children from low-income families, and children whose parents are subsistence farmers, as these children are more vulnerable to falling behind in the early and middle grades. Future research should investigate the mechanisms that underpin the association between breastfeeding and educational outcomes, as well as sex differences and possible mediators. More studies on the subject are warranted in sub-Saharan Africa to contribute to the growing body of literature and broaden understanding of the educational benefits of breastfeeding in the region.

One notable limitation of our study is that only a small number of children were assessed at ages 12 and 13. These small numbers could have reduced the statistical power to detect a difference in older children at the terminal grades. Our analysis did not account for birthweight and gestational age. However, this concern is minimal given that we controlled for known key confounders like parental education, family income, and parental occupation. Since data on exclusive breastfeeding duration were based on maternal recall, we cannot completely rule out the possibility of overreporting due to recall and social desirability bias. However, given that the data were collected in the first 3 months after birth and updated only a few months after the recommended duration for exclusive breastfeeding, any such effects should be minimal. Indeed, our estimated prevalence of exclusive breastfeeding at 6 months is comparable to the 34% reported in the 2015 Malawi Demographic and Health Survey^64^ and the 40% reported by Chipojola et al.^65^. Furthermore, it has been shown that, within the first three years after birth, maternal recall is a valid and reliable method for estimating infant breastfeeding^66^.

## Conclusions

Our findings suggest that a longer duration of exclusive breastfeeding during infancy may promote better grade progression in primary school and decrease the likelihood of being over-age for grade. This finding supports the World Health Organization’s recommendation on exclusive breastfeeding by adding to the growing body of evidence that the benefits of exclusive breastfeeding for 6 months extend beyond the known improvements in infant health and survival. It also suggests that the beneficial effects on educational attainment reported in high-income settings are not entirely due to socioeconomic confounding. Policies encouraging exclusive breastfeeding as the primary feeding method for the first 6 months of life may help improve children's and adolescents’ educational attainment. We encourage more research into the educational benefits of breastfeeding and its possible mechanisms in sub-Saharan Africa to inform infant feeding recommendations, as the literature on the subject from the region is still sparse.

## Supplementary Information

Supplementary Information.

## Data Availability

The data supporting this study's findings are available from the Karonga Health and Demographic Surveillance System in Malawi. However, restrictions apply to the availability of these data, which were used under license for the current study and are not publicly available. The data are, however, available from the corresponding author (SM) upon reasonable request and with permission of the director of the Karonga Health and Demographic Surveillance System in Malawi.
