# Supplementary material for: Effects of exclusive breastfeeding on educational attainment and longitudinal trajectories of grade progression among children in a 13-year follow-up study in Malawi
Source: Sci Rep. 2023 Jul 14;13:11413. doi: 10.1038/s41598-023-38455-5 (PMC10349128; doi:10.1038/s41598-023-38455-5)
Supplement: Supplementary file 1 — Supplementary Information. [file 41598_2023_38455_MOESM1_ESM.docx]

**Effects of exclusive breastfeeding on educational attainment and longitudinal trajectories of grade progression among children in a 13-year follow-up study in Malawi**

Shamsudeen Mohammed^1*^, Emily L Webb^2^, Clara Calvert^3,4^, Judith R Glynn^2^, Bindu S. Sunny^5^, Amelia C. Crampin^2,6,7^, Estelle McLean^4,6^, Shekinah Munthali-Mkandawire^6^, Albert Lazarous Nkhata Dube^6^, Fredrick Kalobekamo^6^, Milly Marston^4**^, and Laura L Oakley^1,8**^

^1^Department of Non-communicable Disease Epidemiology, Faculty of Epidemiology and Population Health, London School of Hygiene & Tropical Medicine, London, UK

^2^Department of Infectious Disease Epidemiology, Faculty of Epidemiology and Population Health, London School of Hygiene & Tropical Medicine, London, UK,

^3^Centre for Global Health, Usher Institute, University of Edinburgh, Edinburgh, UK

^4^Department of Population Health, Faculty of Epidemiology and Population Health, London School of Hygiene & Tropical Medicine, London, UK,

^5^United Nations Educational, Scientific and Cultural Organization, Paris, France

^6^Malawi Epidemiology and Intervention Research Unit, Lilongwe, Malawi,

^7^Institute of Health and Wellbeing, University of Glasgow, Glasgow, United Kingdom

^8^Centre for Fertility and Health, Norwegian Institute of Public Health, Oslo, Norway.

**Joint senior authors

**SUPPLEMENTARY MATERIALS**

**Supplementary Table 1. Multiple imputation models with the number of missing and imputed cases**

|  | **Missing** | **Imputed** | **Imputation model** |
| --- | --- | --- | --- |
|  |  |  |  |
| **Household wealth at birth** | 29 (2.8%) | 29 | Ordered logistic regression |
| **Birth order of child** | 38 (3.7%) | 38 | Ordered logistic regression |
| **Mother education** | 3 (0.3%) | 3 | Ordered logistic regression |
| **Father education** | 51 (5.0%) | 51 | Ordered logistic regression |
| **Father occupation** | 55 (5.4%) | 55 | Multinomial logistic regression |
| **Mother occupation** | 3 (0.3%) | 3 | Multinomial logistic regression |

**Supplementary Table 2 Goodness of fit statistics and model adequacy diagnostics for LCGM**

| **Latent Model** | **Bayesian information criteria (BIC)** | **Akaike Information Criterion (AIC)** | **Entropy** | **Number of latent classes** | **Average Posterior Probability of Assignment (APPA)** | **Number per class** | **% of participants per class** |
| --- | --- | --- | --- | --- | --- | --- | --- |
|  |  |  |  |  |  |  |  |
| 1-class model | -2258.25 | -2248.54 | - |  |  |  |  |
|  |  |  |  | 1 | - | - | - |
| 2-class model | -1620.60 | -1606.02 | 0.85 |  |  |  |  |
|  |  |  |  | 1 | 0.94 | 522 | 54.8 |
|  |  |  |  | 2 | 0.96 | 430 | 45.2 |
| 3-class model | -1545.64 | -1518.92 | 0.82 |  |  |  |  |
|  |  |  |  | 1 | 0.89 | 194 | 20.4 |
|  |  |  |  | 2 | 0.90 | 282 | 29.6 |
|  |  |  |  | 3 | 0.95 | 476 | 50.0 |
| 4-class model | -1544.69 | -1515.54 | 0.64 |  |  |  |  |
|  |  |  |  | 1 | 0.89 | 193 | 20.3 |
|  |  |  |  | 2 | 0.84 | 273 | 28.7 |
|  |  |  |  | 3 | 0.68 | 256 | 26.9 |
|  |  |  |  | 4 | 0.57 | 230 | 24.1 |
| 5-class model | -1624.22 | -1585.35 | 0.68 |  |  |  |  |
|  |  |  |  | 1 | - | - | - |
|  |  |  |  | 2 | 0.64 | 413 | 43.4 |
|  |  |  |  | 3 | 0.91 | 139 | 14.6 |
|  |  |  |  | 4 | 0.92 | 400 | 42.0 |
|  |  |  |  | 5 | - | - | - |

| **Three-class model** | **Four-class model** |
| --- | --- |
|  |  |
| **Three-class model individual level trajectories**  **** | **Four-class model individual level trajectories**  **** |
| **Supplementary Figure 1: Trajectories of three-class and four-class latent class models and individual level age-for-grade progression** | |

**Supplementary Table 3 Goodness of fit statistics and model adequacy diagnostics of girls for LCGM**

| **Latent Model** | **Bayesian information criteria (BIC)** | **Akaike Information Criterion (AIC)** | **Entropy** | **Number of latent classes** | **Average Posterior Probability of Assignment (APPA)** | **Number per class** | **% of particpants per class** |
| --- | --- | --- | --- | --- | --- | --- | --- |
|  |  |  |  |  |  |  |  |
| 1-class model | -943.39 | -935.17 | - |  |  |  |  |
|  |  |  |  | 1 | - | - | - |
| 2-class model | -680.21 | -667.87 | 0.84 |  |  |  |  |
|  |  |  |  | 1 | 0.95 | 299 | 66.2 |
|  |  |  |  | 2 | 0.95 | 153 | 33.8 |
| 3-class model | -672.33 | -655.87 | 0.66 |  |  |  |  |
|  |  |  |  | 3 | 0.94 | 137 | 30.3 |
|  |  |  |  | 2 | 0.89 | 122 | 27.0 |
|  |  |  |  | 1 | 0.77 | 193 | 42.7 |
| 4-class model | -666.72 | -637.92 | 0.62 |  |  |  |  |
|  |  |  |  | 1 | 0.88 | 62 | 13.7 |
|  |  |  |  | 2 | 0.86 | 113 | 24.9 |
|  |  |  |  | 3 | 0.92 | 118 | 26.0 |
|  |  |  |  | 4 | 0.64 | 160 | 35.4 |
|  |  |  |  |  |  |  |  |
| 5-class model | -696.21 | -663.30 | 0.70 |  |  |  |  |
|  |  |  |  | 1 | - | - | - |
|  |  |  |  | 2 | 0.94 | 132 | 29.2 |
|  |  |  |  | 3 | 0.89 | 70 | 15.5 |
|  |  |  |  | 4 | 0.76 | 250 | 55.3 |
|  |  |  |  | 5 | - | - | - |

| **Three-class model** | **Four-class model** |
| --- | --- |
|  |  |
| **Three-class model individual level trajectories**  **** | **Four-class model individual level trajectories**  **** |
| **Supplementary Figure 2: Trajectories of three-class and four-class latent class models and individual level age-for-grade progression of girls** | |

**Supplementary Table 4 Goodness of fit statistics and model adequacy diagnostics of boys for LCGM**

| **Latent Model** | **Bayesian information criteria (BIC)** | **Akaike Information Criterion (AIC)** | **Entropy** | **Number of latent classes** | **Average Posterior Probability of Assignment (APPA)** | **Number per class** | **% of participants per class** |
| --- | --- | --- | --- | --- | --- | --- | --- |
|  |  |  |  |  |  |  |  |
| 1-class model | -1269.15 | -1260.72 | - |  |  |  |  |
|  |  |  |  | 1 | - | - | - |
| 2-class model | -935.31 | -922.67 | 0.86 |  |  |  |  |
|  |  |  |  | 1 | 0.95 | 219 | 44.0 |
|  |  |  |  | 2 | 0.95 | 281 | 56.0 |
| 3-class model | -921.59 | -898.41 | 0.81 |  |  |  |  |
|  |  |  |  | 2 | 0.93 | 142 | 28.4 |
|  |  |  |  | 1 | 0.91 | 198 | 39.6 |
|  |  |  |  | 3 | 0.93 | 160 | 32.0 |
| 4-class model | -896.20 | -870.91 | 0.68 |  |  |  |  |
|  |  |  |  | 1 | 0.90 | 135 | 27.0 |
|  |  |  |  | 2 | 0.85 | 158 | 31.6 |
|  |  |  |  | 3 | 0.65 | 136 | 27.2 |
|  |  |  |  | 4 | 0.71 | 71 | 14.2 |
| 5-class model | -958.88 | -918.84 | 0.78 |  |  |  |  |
|  |  |  |  | 1 | - | - | - |
|  |  |  |  | 2 | 0.93 | 255 | 48.7 |
|  |  |  |  | 3 | - | - | - |
|  |  |  |  | 4 | 0.79 | 173 | 27.8 |
|  |  |  |  | 5 | 0.92 | 72 | 23.5 |

| **Three-class model** | **Four-class model** |
| --- | --- |
|  |  |
| **Three-class model individual level trajectories**  **** | **Four-class model individual level trajectories**  **** |
| **Supplementary Figure 3: Trajectories of three-class and four-class latent class models and individual level age-for-grade progression of boys** | |

**Supplementary Table 5 Binary logistic regression analysis of the association between exclusive breastfeeding duration and age-for-grade at age 11.5 (mid-point of age 10-12) in Malawi (Complete case analysis)**

|  | **N** | **Age-for-grade** | | **Unadjusted odds ratio (95% CI)** | **P-value** | **Adjusted odds ratio (95% CI)** | **P-value** |
| --- | --- | --- | --- | --- | --- | --- | --- |
|  |  | **Underage or on-time for grade** | **Overage for grade** |  |  |  |  |
|  |  | **n (%)** | **n (%)** |  |  |  |  |
| **Model 1 Both sexes: Duration of exclusive breastfeeding (n=784)** | | | |  |  |  |  |
| 0-2 months | 144 | 68 (47.2) | 76 (52.8) | 1.00 | 0.65 | 1.00 | 0.97 |
| 3-5 months | 355 | 177 (49.9) | 178 (50.1) | 0.90 (0.61 - 1.33) |  | 1.04 (0.68 - 1.58) |  |
| 6 months | 285 | 148 (51.9) | 137 (48.1) | 0.83 (0.55 - 1.24) |  | 1.06 (0.68 - 1.64) |  |
| **Model 2 Girls: Girls (n=375): Duration of exclusive breastfeeding** | | | | |  |  |  |
| 0-2 months | 62 | 38 (61.3) | 24 (38.7) | 1.00 | 0.89 | 1.00 | 0.50 |
| 3-5 months | 175 | 105 (60.0) | 70 (40.0) | 1.06 (0.58 - 1.91) |  | 1.05 (0.55 - 2.02) |  |
| 6 months | 138 | 80 (58.0) | 58 (42.0) | 1.15 (0.62 - 2.12) |  | 1.38 (0.71 - 2.69) |  |
| **Model 3 Boys: Boys (403): Duration of exclusive breastfeeding** | | | | |  |  |  |
| 0-2 months | 82 | 30 (36.6) | 52 (63.4) | 1.00 | 0.37 | 1.00 | 0.63 |
| 3-5 months | 177 | 69 (39.0) | 108 (61.0) | 0.90 (0.53 - 1.55) |  | 1.12 (0.63 - 2.02) |  |
| 6 months | 144 | 65 (45.1) | 79 (54.9) | 0.70 (0.40 - 1.22) |  | 0.89 (0.48 - 1.63) |  |

Note: We controlled for child's sex, household wealth at birth, age of mother at birth, birth order of child, maternal HIV status, mother education, father education, mother occupation, father occupation, and distance to a tarmac road in the adjusted analysis.

**Supplementary Table 6. Generalised estimating equations analysis of the effects of exclusive breastfeeding duration on age-for-grade among school-aged children in Malawi (Complete case analysis)**

|  | **Unadjusted odds ratio (95% CI)** | **P-value** | **Adjusted odds ratio (95% CI)** | **P-value** |
| --- | --- | --- | --- | --- |
|  |  |  |  |  |
| **Model 1 Both sex and all ages: Duration of exclusive breastfeeding (n=867)** | | |  |  |
| 0-2 months | 1.00 | 0.01* | 1.00 | 0.13* |
| 3-5 months | 0.90 (0.69 - 1.17) |  | 0.99 (0.77 - 1.28) |  |
| 6 months | 0.71 (0.54 - 0.94) |  | 0.83 (0.64 -1.09) |  |
| **Model 2 Age 6-9 Bothe sexes: Age 6-9: Duration of exclusive breastfeeding (n=857)** | | |  |  |
| 0-2 months | 1.00 | 0.001* | 1.00 | 0.02* |
| 3-5 months | 0.86 (0.59 - 1.26) |  | 0.98 (0.67 - 1.43) |  |
| 6 months | 0.52 (0.34 - 0.80) |  | 0.63 (0.42 - 0.97) |  |
| **Model 3 Age 10-13 Both sexes: Age 10-13: Duration of exclusive breastfeeding (n=788)** | | | |  |
| 0-2 months | 1.00 | 0.30 | 1.00 | 0.86 |
| 3-5 months | 0.90 (0.63 - 1.29) |  | 1.04 (0.72 - 1.51) |  |
| 6 months | 0.76 (0.53 - 1.11) |  | 0.95 (0.65 - 1.41) |  |
| **Model 4 Girls, all ages: Girls: Duration of exclusive breastfeeding (n=425)** | | |  |  |
| 0-2 months | 1.00 | 0.22 | 1.00 | 0.48 |
| 3-5 months | 1.04 (0.66 - 1.63) |  | 1.04 (0.66 - 1.65) |  |
| 6 months | 0.77 (0.49 - 1.23) |  | 0.85 (0.53 - 1.36) |  |
| **Model 5 Boys, all ages: Boys: Duration of exclusive breastfeeding (n=443)** | | |  |  |
| 0-2 months | 1.00 | 0.14 | 1.00 | 0.44 |
| 3-5 months | 0.85 (0.62 - 1.17) |  | 0.99 (0.73 - 1.34) |  |
| 6 months | 0.71 (0.51 - 1.00) |  | 0.84 (0.61 - 1.16) |  |

*Test for linear trend

Note: We controlled for child's sex, household wealth at birth, age of mother at birth, birth order of child, maternal HIV status, mother education, father education, mother occupation, father occupation, and distance to a tarmac road in the adjusted analysis.

**Supplementary Table 7 Multinomial logistic regression analysis of the association between exclusive breastfeeding duration and age-for-grade trajectories among school-aged children in Malawi (Complete case analysis)**

|  | **Falling behind from early grades vs consistently on time for grade** | | **Falling behind from middle grades vs consistently on time for grade** | | **Falling behind in terminal grades vs consistently on time for grade** | |
| --- | --- | --- | --- | --- | --- | --- |
|  | **OR (95% CI)** | **aOR (95% CI)** | **OR (95% CI)** | **aOR (95% CI)** | **OR (95% CI)** | **aOR (95% CI)** |
| **Model 1 Both sexes and all ages: Duration of exclusive breastfeeding (n=812)** | | | | |  |  |
| 0-2 months | 1.00 | 1.00 | 1.00 | 1.00 | 1.00 | 1.00 |
| 3-5 months | 0.89 (0.55 - 1.42) | 1.06 (0.65 - 1.74) | 1.05 (0.68 - 1.61) | 1.17 (0.75 - 1.82) | 1.12 (0.89 - 1.42) | 1.14 (0.90 - 1.45) |
| 6 months | 0.53 (0.32 - 0.87) | 0.69 (0.41 - 1.16) | 0.91 (0.58 - 1.41) | 1.13 (0.71 - 1.78) | 0.94 (0.75 - 1.17) | 0.95 (0.76 - 1.19) |
| **Model 2 Girls, all ages: Girls: Duration of exclusive breastfeeding (n=329)** | | | | |  |  |
| 0-2 months | 1.00 | 1.00 | 1.00 | 1.00 | 1.00 | 1.00 |
| 3-5 months | 1.15 (0.54 - 2.45) | 1.21 (0.53 - 2.78) | 1.33 (0.69 - 2.56) | 1.25 (0.63 - 2.52) | 1.25 (0.88 - 1.78) | 1.25 (0.87 - 1.79) |
| 6 months | 0.67 (0.30 - 1.49) | 0.76 (0.33 - 1.78) | 1.40 (0.73 - 2.69) | 1.53 (0.76 - 3.07) | 1.08 (0.77 - 1.52) | 1.08 (0.77 - 1.52) |
| **Model 3 Boys, all ages: Boys: Duration of exclusive breastfeeding (n=420)** | | | | |  |  |
| 0-2 months | 1.00 | 1.00 | 1.00 | 1.00 | 1.00 | 1.00 |
| 3-5 months | 0.76 (0.40 - 1.45) | 1.01 (0.51 - 2.00) | 0.89 (0.48 - 1.65) | 1.13 (0.59 - 2.19) | 1.00 (0.73 - 1.37) | 1.03 (0.75 - 1.42) |
| 6 months | 0.42 (0.21 - 0.82) | 0.55 (0.27 - 1.11) | 0.62 (0.33 - 1.17) | 0.84 (0.43 - 1.66) | 0.81 (0.60 - 1.09) | 0.83 (0.62 - 1.12) |

Note: We controlled for child's sex, household wealth at birth, age of mother at birth, birth order of child, maternal HIV status, mother education, father education, mother occupation, father occupation, and distance to a tarmac road in the adjusted analysis.
